# Does risperidone seem safe in patients with antipsychotic-induced leukoneutropenia? Case report

**DOI:** 10.1192/j.eurpsy.2023.1235

**Published:** 2023-07-19

**Authors:** W. Abid, F. Chérif, H. Trigui, S. Hentati, I. Feki, R. Sallemi, J. Masmoudi

**Affiliations:** psychiatry “A” department, hedi Chaker University Hospital, sfax, Tunisia

## Abstract

**Introduction:**

Antipsychotic-related hematological abnormalities have been reported in the literature, but remain a rare complication of some second-generation antipsychotic drugs. It has been suggested that risperidone is the preferred alternative when adverse hematological effects have been induced by conventional antipsychotic drugs. Blood dyscrasia adverse reactions have been reported rarely with risperidone.

**Objectives:**

We aims to call attention to leukopenia as a potential side effect of during treatment with risperidone.

**Methods:**

We report a case in which a patient developed leukoneutropenia during treatment with risperidone

**Results:**

A 46-year-old man was admitted to psychiatric department for aggressive behavior. The patient had a history of right bundle branch block and has been diagnosed with schizophrenia at age of 36 years. This patient had only one psychiatric hospitalization in 2021 where he developed persistent leuko-neutropenia. A viral cause was retained. The patient was discharged on risperidone 4 mg and had been poorly compliant with his antipsychotic medication for the last year.

Currently, the patient was excited, very talkative and refused medication. He received an intramuscular injection of haloperidol 10 mg and chlorpromazine 50 mg. Under these doses, the patient became sedated and his balance sheet showed a leukoneuropenia amounting to 1960 cells/mm3. Then, it was decided to stop chlorpromazine and Haloperidol and put the patient on Diazepam. A complete blood count was done the next day showing that his white Blood Cells (WBC) count went up to 4360 cells /mm3 (neutrophils rate = 62,4 %). The reintroduction of haloperidol with diazepam caused the WBC to fall back to 2000. Haloperidol stopped as a possible cause of the leucopenia. The patient started taking risperidone orally 1 mg daily, which was gradually up titrated to 4 mg daily. Two weeks later the WBC went up to 4680 cells/mm3 (neutrophil rate = 60,4%) three week after stopping haloperidol. However, three days after increasing the dose of risperidone to 5 mg the leukoneutropenia recurred, (WBC = 2960 cells/mm3, neutrophil rate = 41%). When risperidone was reduced to 4 mg, his WBC count remained stable for two weeks (WBC = 2970, neutrophil rate = 48,9%). Clinically , the patient is no longer excited.

**Conclusions:**

Risperidone-induced leuko-neutropenia (RILN) is very unusual and its incidence rate is unknown. Currently, there are no evidence-based alternative antipsychotic recommandations for RILN. For the case presented here, we achieved stabilisation of RILN by dose reduction.

Routine monitoring of the WBC level of patients on risperidone treatment, regardless of their hematological baseline, might be good practice for all psychiatrists. We recommend extending this practice to inpatient and outpatient services.

**Disclosure of Interest:**

None Declared

